# Thermal Percolation Behavior in Thermal Conductivity of Polymer Nanocomposite with Lateral Size of Graphene Nanoplatelet

**DOI:** 10.3390/polym14020323

**Published:** 2022-01-13

**Authors:** Ji-un Jang, Hae Eun Nam, Soon Oh So, Hyeseong Lee, Geon Su Kim, Seong Yun Kim, Seong Hun Kim

**Affiliations:** 1 Department of Organic and Nano Engineering, Hanyang University, 222 Wangsimni-ro, Haengdang-dong, Seongdong-gu, Seoul 04763, Korea; jju204@hanyang.ac.kr; 2 Division of Polymer-Nano & Textile Engineering, Jeonbuk National University, 567 Baekje-daero, Deokjin-gu, Jeonju-si 54896, Jeonbuk, Korea; hae_eun1912@naver.com (H.E.N.); tnsdh0626@naver.com (S.O.S.); dlgptjd6715@naver.com (H.L.); kuean88@naver.com (G.S.K.)

**Keywords:** composite, graphene nanoplatelet, thermal conductivity, thermal percolation

## Abstract

In this study, the thermal percolation behavior for the thermal conductivity of nanocomposites according to the lateral size of graphene nanoplatelets (GNPs) was studied. When the amount of GNPs reached the critical concentration, a rapid increase in thermal conductivity and thermal percolation behavior of the nanocomposites were induced by the GNP network. Interestingly, as the size of GNPs increased, higher thermal conductivity and a lower percolation threshold were observed. The in-plane thermal conductivity of the nanocomposite containing 30 wt.% M25 GNP (the largest size) was 8.094 W/m·K, and it was improved by 1518.8% compared to the polymer matrix. These experimentally obtained thermal conductivity results for below and above the critical content were theoretically explained by applying Nan’s model and the percolation model, respectively, in relation to the GNP size. The thermal percolation behavior according to the GNP size identified in this study can provide insight into the design of nanocomposite materials with excellent heat dissipation properties.

## 1. Introduction

Various efforts are in progress to realize the excellent thermal conductivity of nanocarbon in practical applications [1,2,3,4]. It is well known that the thermal conductivity of nanocarbon, represented by carbon nanotubes (CNTs, ~3000 W/m·K [5]) and graphene (~5000 W/m·K [6]), is the best among existing materials. However, utilizing the excellent thermal conductivity of nanocarbon in practical applications is limited by the difficulty in handling it because of its nanoscale size [7,8]. To overcome this handling challenge and, at the same time, to take advantage of its light weight, excellent processability, and shape stability, the interest in nanocarbon-based polymer composites continues to grow.

It is known that the electrical conductivity of a polymer composite filled with nanocarbon is well explained by percolation behavior based on the electron tunneling effect, indicating that the electrical conductivity is rapidly improved at a certain filler content [9]. Given this background, inducing the uniform dispersion of conductive fillers to maximize the electron tunneling effect is regarded as the most important physical factor for improving the electrical conductivity of polymer composites. On the other hand, since the thermal conductivity of a polymer composite filled with nanocarbon is dominated by phonon transfer rather than the movement of electrons, the interfacial thermal resistance (ITR) caused by phonon scattering due to lattice mismatch between the nanocarbon filler and the polymer matrix is recognized as the most important physical factor causing the decrease in the thermal conductivity of polymer composites [10,11]. Kim et al. [12] reported that as the length of multi-walled CNTs increased, the thermal conductivity of the nanocomposite was improved due to a reduction in the ITR effect caused by a decrease in phonon scattering at the end of the filler. In addition, the authors [13] found that as the lateral size or thickness of graphene nanoplatelets (GNPs) increased, the thermal conductivity of the nanocomposite was improved because phonon scattering was reduced due to the decrease in the interface between the filler and polymer matrix [14,15]. Therefore, it can be confirmed that controlling the ITR is important for optimizing the thermal conductivity of a nanocarbon-filled polymer composite, and as the region where the shape of the filler meets the polymer matrix is reduced, the ITR effect decreases, resulting in excellent thermal conductivity.

There has been a debate about whether percolation behavior appears in the thermal conductivity of nanocarbon composites [16,17,18,19]. Shtein et al. [16] reported that the thermal percolation behavior, defined as the rapid improvement of thermal conductivity above a certain content, was caused by the overlapping of GNPs (GNP to GNP contact) within the polymer matrix. Similarly, Kim et al. [17] induced the thermal percolation behavior of polymer composites by ensuring contact between fillers by controlling the expansion degree of expanded graphite. The authors identified that this behavior was due to the conversion from the ITR-dominant system to a system dominated by contact thermal resistance, which is the result of phonon scattering at inter-filler contacts. Recently, Barani et al. [18] reported the thermal percolation behavior resulting from contact between fillers in a hybrid filler system that simultaneously incorporated two or more fillers.

In this study, in order to minimize the effect of ITR on the thermal conductivity of polymer composites, thermal percolation behavior according to the lateral size and content of GNPs was investigated. A two-step process consisting of masterbatch formation and melt compounding was applied. Masterbatch formation was performed based on an in situ polymerizable, low-viscosity oligomeric matrix to maximize the contact between fillers while simultaneously inducing uniform filler dispersion. The thermal conductivity of the prepared GNP-containing nanocomposite was analyzed according to the lateral size and content of GNPs, and it was observed that as the lateral size of GNPs increased, the thermal conductivity increased due to a decrease in the ITR effect. In addition, it was confirmed that the thermal percolation threshold shifted to the low-content portion as the lateral size of GNPs increased. This behavior was theoretically investigated based on Nan’s model and the percolation model.

## 2. Experimental Methods

### 2.1. Materials

As shown in Figure S1 of Supplementary Materials, GNPs (M5, M15, and M25; XG Science, Lansing, MI, USA) with similar thickness and various lateral sizes (about 5, 15, and 20 μm) were used as conductive fillers to improve the thermal conductivity of nanocomposites (the characterization of GNPs is presented in Figure S2) [13,20]. Cyclic butylene terephthalate (CBT; CBT 160, Cyclics^®^ Co., Schenectady, NY, USA) was selected for the matrix of the composite. CBT is an oligomeric matrix composed of 2–7 cyclic oligoesters. It melts at a temperature in the range of 130–150 °C and exhibits a low melt viscosity of about 0.02 Pa·s, which is known to be advantageous for dispersing nanofillers [21,22]. In addition, when processed at a temperature of 160 °C or higher, it is polymerized in situ and converted into polymerized CBT (pCBT), which has a molecular structure similar to that of linear polybutylene terephthalate.

### 2.2. Fabrication

**Figure 1:**
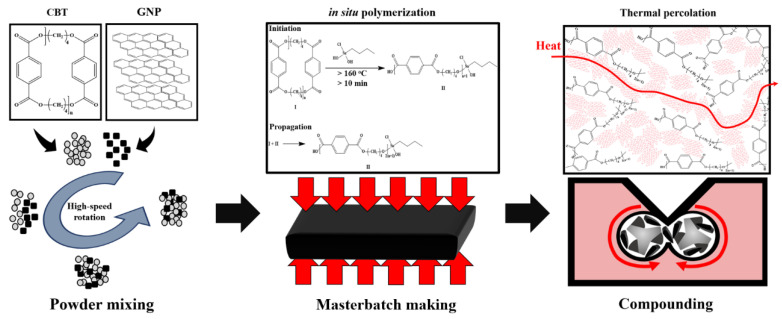
Schematic of composite fabrication using the proposed process.

To remove moisture, the prepared CBT and GNP powders were stored in a desiccator at a moisture content of 0.5% or less for 12 h. The pulverized resin and filler were weighed to obtain the target concentrations in Table 1 and then mixed at a high rotation speed (2000 rpm) for 2 min using a Thinky mixer (ARE 310, Thinky Co., Tokyo, Japan), as shown in Figure 1. The mixture was compressed in the form of a masterbatch at 230 °C for 3 min with a pressure of 15 MPa using a hot press (D3P-20J, DaeHeung Science, Incheon, Korea) and then compounded using an internal mixer (HAAKE^TM^ Rheomix 600R OS Mixer, Thermo Scientific Inc., Marietta, GA, USA) at 230 °C and 60 rpm. The compound was hot-pressed again using a mold of 25 × 25 × 2 mm^3^ for characterization.

### 2.3. Characterization

The thermal conductivity of the prepared composite was measured by the hot-disk method (ISO 22007-2 standard) using a thermal conductivity analyzer (TPS 2500 S, Hot Disk AB, Gothenburg, Sweden). A sensor composed of a double helical nickel wire was placed at the center of the specimens, and the thermal resistance value was determined by measuring the temperature change due to the electric power provided by the heat source. The thermal resistance values based on power and temperature changes were calculated to determine the thermal conductivity of the composite by substituting it into the Fourier equation using software. To confirm the mechanism of thermal conductivity improvement by the incorporated nanocarbon filler, the prepared composite was fractured after freezing with liquid nitrogen, and the fracture surface was coated with Pt for 100 sec using a sputter instrument (EM ACE 200, Leica Microsystems, Wetzlar, Germany). The prepared sample was observed under a voltage of 15 kV using a field-emission scanning electron microscope (FE-SEM; GeminiSEM 500, Zeiss, Oberkrochen, Germany). Micro-computed tomography (micro-CT; Skyscan 1172, Bruker Co., Billerica, MA, USA) with an X-ray source of tungsten was used for nondestructive three-dimensional (3D) analysis applied to a large area (~1 mm^3^). The specimen was cut to a size of 2 × 2 × 2 mm^3^, and then images were captured with a resolution of >0.7 μm at a voltage of 15 V. The raw images of the specimen obtained by irradiating X-rays while rotating by 0.2° were reconstructed into a 3D structure using software. A thermal image of the prepared composite was analyzed using an infrared imaging camera (FLUKE Ti200, Fluke Co., Ltd., Everett, Washington, DC, USA) with a resolution of 0.075 K. The temperature at the center point of the composite was measured by placing the specimen on a hot plate at 100 °C, recording the value after 2.5 s.

### 2.4. Theoretical Calculations

Nan’s model is a useful equation to estimate the thermal conductivity of nanocarbon filler composites and assumes perfect filler dispersion and a filler–matrix interface [23]. Each filler is wrapped by a polymer layer within the composite and constitutes a Kapitza radius due to the thermal resistance generated between them. The Kapitza radius is related to ITR and is specified as a factor that interferes with the phonon transfer in the composite [23]. The thermal percolation behavior of a composite incorporating graphene exceeding its critical volume fraction was reported [16]. The thermal conductivity of the composite, which was gradually increased by ITR in the specimen with low filler content, showed a dramatic enhancement in the filler fraction above the critical percolation threshold ($\phi_{c}$). The filler contact formed in the composite was considered the major factor contributing to the dramatic enhancement of the thermal conductivity. This means that a high content of nanocarbon increases the filler contact in the composite and induces an excellent heat conduction pathway between fillers, which leads to a reduction in phonon scattering because of the ITR at the polymer–filler interface [16,17,19]. Moreover, because the heat transfer mechanism has changed, the improved thermal conductivity of the composite with filler content above the critical percolation threshold can be evaluated by a percolation equation including the pre-exponential factor ($TC_{o}$) and the exponent (t) [16,19].

#### 2.4.1. Nan’s Model

The thermal conductivity of a composite incorporating uniformly dispersed fillers can be predicted by Nan’s model [23]. The theoretical thermal conductivity ($TC_{Nan}$) of the composite based on the ITR (≈Kapitza radius) from the interface between the polymer and nanocarbon filler is expressed as follows:

(1) $$TC_{Nan}=TC_{M}\times \left(\frac{3+\phi_{GNP}\times \left(\beta_{x}+\beta_{z}\right)}{3-\phi_{GNP}\times \beta_{x}}\right)$$

where:

(2) $$\beta_{x}=\frac{2\left(K_{11}^{C}-TC_{M}\right)}{K_{11}^{C}+TC_{M}},\;\beta_{z}=\frac{K_{33}^{C}}{TC_{M}}-1$$

$TC_{M}$ is the thermal conductivity of the matrix (0.5 W/m·K), and $\phi_{GNP}$ denotes the volume fraction of GNPs. $K_{11}^{C}$ and $K_{33}^{C}$ represent equivalent thermal conductivities of GNPs coated with horizontal and vertical interfacial thermal barrier layers of the unit cell, respectively, and can be expressed as follows:

(3) $$K_{11}^{C}=\frac{TC_{GNP}}{1+\frac{2a_{k}TC_{GNP}}{hTC_{M}}},\;K_{33}^{C}=\frac{TC_{GNP}}{1+\frac{2a_{k}TC_{GNP}}{dTC_{M}}},\;a_{k}=R_{ITR}\times TC_{M}$$

where $TC_{GNP}$, $a_{k}$, and $R_{ITR}$ are the thermal conductivity of the GNP (3000 W/m·K), Kapitza radius (40 nm), and ITR (9 × 10^−8^ m^2^ K/W), respectively [24], and h and d denote the thickness (7 nm) and lateral size (5, 15, and 20 μm) of the GNP, separately.

#### 2.4.2. Percolation Equation

As the filler content increases, the filler contact area within the composite becomes a major factor. Evaluation of thermal conductivity while accounting for connected fillers can be performed using the percolation equation. The main factor in the thermal conductivity of composites shifts from ITR to the percolated filler network due to contact between fillers. The thermal conductivity of the composite at the content above the percolated filler network ($TC_{P}$) is expressed as follows:

(4) $$TC_{P}=TC_{M}\times \left(1-\phi_{GNP}\right)+TC_{o}{\left(\frac{\phi_{G}-\phi_{c}}{1-\phi_{c}}\right)}^{t}$$

where $TC_{P}$, $TC_{M}$ and $\phi_{GNP}$ are the theoretical conductivity of a composite filled with a thermally percolated filler network, the thermal conductivity of the matrix (0.5 W/m·K), and the volume fraction of GNPs, respectively. In this study, $\phi_{c}$ was 0.16 (16 vol.%), 0.13 (13 vol.%), and 0.062 (6.2 vol.%) for M5, M15, and M25, respectively. $TC_{o}$ and t are the pre-exponential factor (65 W/m·K in this study) and critical exponent (1.01 in this study) [16,17].

## 3. Results and Discussion

**Figure 2:**
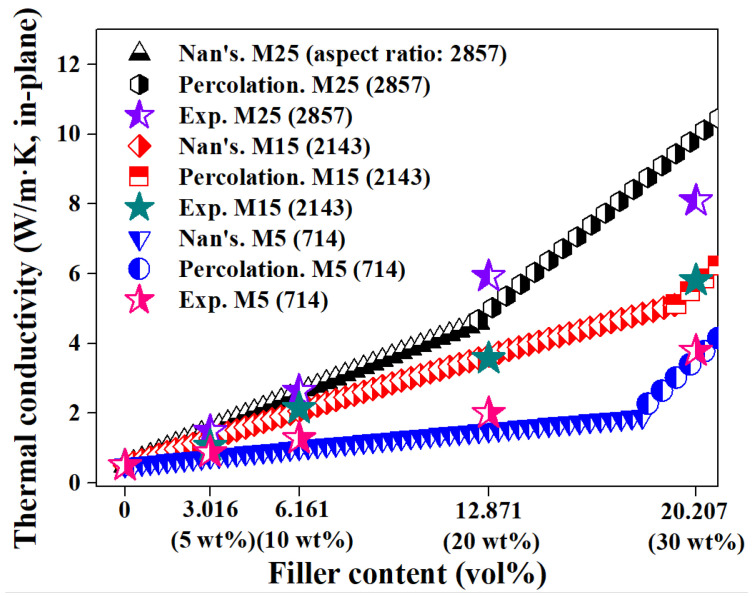
Experimental and theoretical thermal conductivity of the fabricated composites.

The thermal conductivity of the prepared nanocomposite is shown in Figure 2. The horizontal ordinate is the converted volume fraction of the filler in the fabricated composite, and the number in parentheses indicates the mass fraction. Regardless of the lateral size of GNPs, a rapid improvement in thermal conductivity was observed above each specific concentration (M5 30 wt.%, M15 30 wt.%, M25 20 and 30 wt.%). This thermal percolation behavior was determined to be due to the direct contact between fillers with the increase in the incorporated GNP content. In addition, as the lateral size of the incorporated GNPs increased, the experimentally measured thermal conductivity of the nanocomposite was improved. This can be explained by comparing the content before and after the rapid thermal conductivity improvement. First, when the critical content was not reached, the interface between the GNP filler and the pCBT matrix became smaller as the lateral size of the GNP increased and resulted in the reduction in ITR [17]. Therefore, the thermal conductivities of M5 20 wt.%, M15 20 wt.%, and M25 10 wt.% were improved to 2.002 (300.4% improvement compared to the neat pCBT), 3.570 (614.0%), and 2.637 W/m·K (427.4%) below the critical content ($\phi_{c}$ = 16, 13, and 6.2 vol.%, corresponding to M5, M15, and M25, respectively). Second, in the case of the critical content or above, the contact between fillers was appropriately induced, and the formation of a connected filler network was activated as the lateral size of the GNP increased (see Figure S3). As a result of these networks, enhanced thermal conductivities (3.788, 5.807, 5.922, and 8.094 W/m·K for M5 30 wt.%, M15 30 wt.%, and M25 20 and 30 wt.%, respectively) were observed, with increases of 657.6%, 1061.4%, 1084.4%, and 1518.8% compared to the neat pCBT. Table S1 summarizes the in-plane thermal conductivities of composites with GNP fillers reported in previous studies [13,22,25,26,27,28].

The theoretical evaluation for the measured thermal conductivity of composites can also be divided into below and above the critical content of nanocarbon filler. Below the critical content, it is reasonable to apply Nan’s model, which assumes uniform dispersion without filler contact, because the contact between GNPs is minimized based on the uniform GNP dispersion induced by the applied process. As shown in Figure 2, it was confirmed that the calculated results of Nan’s model and the experimental results were in good agreement regardless of the type of GNP, indicating that the GNPs incorporated in the fabricated nanocomposite were uniformly dispersed without contact. In the case of the critical content or above, the percolation model was used for the evaluation since contact between GNPs occurred, forming a connected network. The experimental thermal conductivity above the critical content showed a significant error in the theoretical result of Nan’s model but was in good agreement with the theoretical result of the percolation model. Based on these results, the formation of a connected filler network was expected to be induced by physical contact between GNPs above the critical content.

**Figure 3:**
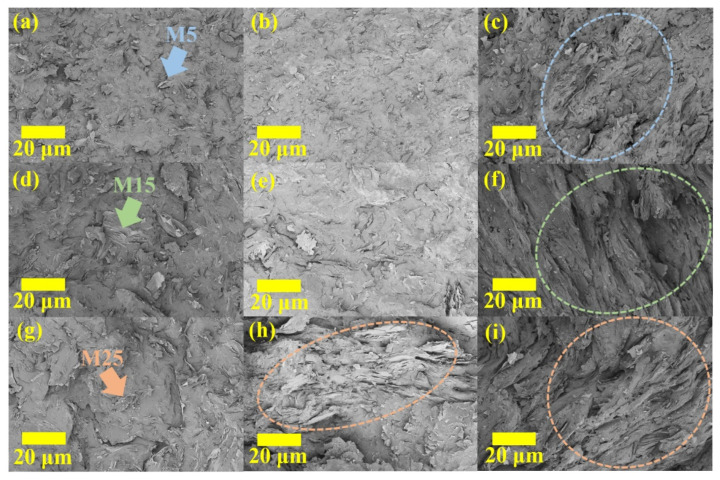
FE-SEM images of the composites with M5 (**a**) 10 wt.%, (**b**) 20 wt.%, and (**c**) 30 wt.%; M15 (**d**) 10 wt.%, (**e**) 20 wt.%, and (**f**) 30 wt.%; and M25 (**g**) 10 wt.%, (**h**) 20 wt.%, and (**i**) 30 wt.%.

**Figure 4:**
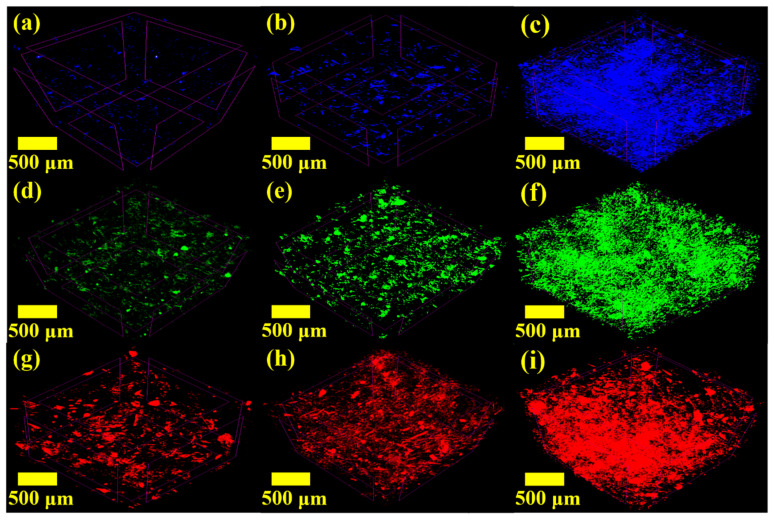
Micro-CT images of the composites with M5 (**a**) 10 wt.%, (**b**) 20 wt.%, and (**c**) 30 wt.%; M15 (**d**) 10 wt.%, (**e**) 20 wt.%, and (**f**) 30 wt.%; and M25 (**g**) 10 wt.%, (**h**) 20 wt.%, and (**i**) 30 wt.%.

Precise internal structure analysis was required to clarify the thermal conductivity results of the composites mentioned above. Figure 3 shows the FE-SEM images of the fracture surfaces of the composites (surface photos of the fabricated specimens are shown in Figure S4). Below the critical content (Figure 3a,b,d,e,g), it was confirmed that the GNPs were uniformly dispersed without contact. These results imply that the fabrication process involving powder mixing and low-viscosity oligomer resin was suitable for the uniform dispersion of nanocarbon fillers [9,19,21]. In addition, since contact between the fillers was barely observed, it was possible to confirm that the thermal conductivity of the composite was dominated by the internal structure and mainly affected by the ITR. On the other hand, above the critical content, networks formed by contact between GNP fillers were observed (Figure 3c,f,h,i). It was determined that the filler contacts reduced phonon scattering occurring at the interface between the polymer and fillers, which is the dominant factor in heat transfer in the nanocomposite, and caused thermal percolation by inducing thermal conduction between the fillers. To confirm the local filler contact observed on the fracture surface of the fabricated composite in a wide area (~1 mm^3^), images taken by nondestructive 3D analysis using micro-CT were captured and are shown in Figure 4. Below the critical content (Figure 4a,b,d,e,g), uniformly dispersed GNPs were clearly observed in 3D, confirming that they exhibited internal structures consistent with the assumption of Nan’s model. On the other hand, above the critical content (Figure 4c,f,h,i), contact between GNPs and the formation of GNP networks were clearly observed (see Video S1). These results mean that proper phonon transfer was induced due to filler contact and network formation in the composite. Therefore, it was confirmed that the morphological results based on FE-SEM and micro-CT analysis supported the experimental and theoretical thermal conductivity of the composites.

**Figure 5:**
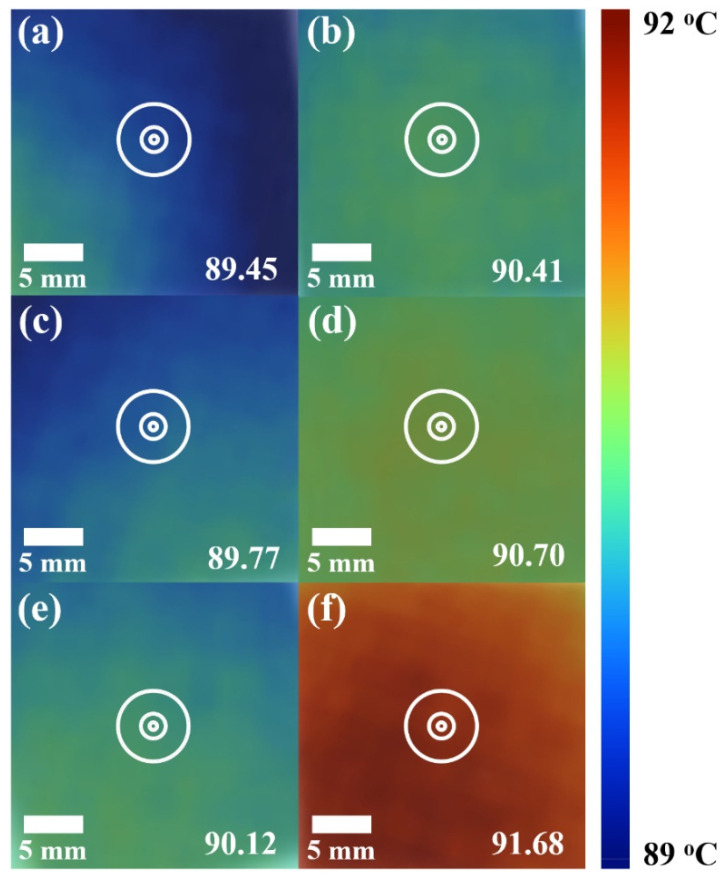
Thermal images of the composites with M5 (**a**) 10 wt.% and (**b**) 30 wt.%; M15 (**c**) 10 wt.% and (**d**) 30 wt.%; and M25 (**e**) 10 wt.% and (**f**) 30 wt.%.

An infrared imaging camera was used to confirm that the experimentally and theoretically evaluated thermal conductivity of the composite can be applied to the practical heat dissipation performance, as shown in Figure 5. Below the critical content (10 wt.% GNPs), higher heat dissipation was observed as the lateral size of the filler increased (89.45, 89.77, and 90.12 °C for M5, M15, and M25, respectively). This result is in good agreement with the thermal conductivity trend of composites dominated by ITR and the theoretically evaluated thermal conductivity using Nan’s model. On the other hand, excellent heat dissipation performance was exhibited above the critical content (30 wt.% GNPs). Filler contacts inside the composite affected the occurrence of thermal percolation and also induced the rapid improvement of thermal conductivity and dramatic enhancement of the heat dissipation performance. In addition, it was confirmed that the larger the lateral size of the GNP, the better the heat dissipation performance (90.41, 90.70, and 91.68 °C for M5, M15, and M25, respectively), which is similar to the experimental and theoretical thermal conductivity results. Therefore, superior heat dissipation performance can be induced by ensuring contact between fillers and establishing a nanocomposite with a structure having excellent heat conduction by incorporating a connected filler network using the proposed process.

## 4. Conclusions

In this study, to reduce the effect of ITR, which is known to be a major factor affecting the thermal conductivity of polymer composites, the thermal percolation behavior according to the lateral size and filler content of GNPs was investigated. A two-step process consisting of masterbatch formation and melt compounding was applied to fabricate polymer composites exhibiting thermal percolation. An in situ polymerizable, low-viscosity CBT matrix was employed to maximize the contact between fillers while simultaneously inducing uniform filler dispersion. Melt compounding contributed to improving the shape stability of the fabricated composites. Below the critical content of GNP fillers, a nanocomposite incorporating uniformly dispersed GNP fillers that were not in contact was prepared. As the lateral size of the GNP increased, the thermal conductivity of the composite was improved due to the major influence of ITR. These thermal conductivity results are in good agreement with the results calculated based on Nan’s model, confirming that an internal structure similar to the assumption of Nan’s model was realized. A rapid improvement in thermal conductivity was observed above the critical content where GNP fillers were in contact to form a connected GNP network. In addition, in the thermal percolation behavior, the larger the lateral size of GNPs, the higher the thermal conductivity of the composite due to the reduction in the ITR effect. The thermal conductivity and the heat dissipation characteristics analyzed by thermal images show good agreement with the amount and lateral size of incorporated GNPs. Based on these results, it was confirmed that excellent heat dissipation performance can be induced by improving the thermal conductivity of the nanocomposite with a connected filler network by controlling the amount and lateral size of two-dimensional fillers.

## Figures and Tables

**Table 1:** Compositions of the fabricated composites.

Sample	GNP			pCBT (wt.%)
	M5 (wt.%)	M15 (wt.%)	M25 (wt.%)	
pCBT	-	-	-	100
M5-pCBT-5	5	-	-	95
M5-pCBT-10	10	-	-	90
M5-pCBT-20	20	-	-	80
M5-pCBT-30	30	-	-	70
M15-pCBT-5	-	5	-	95
M15-pCBT-10	-	10	-	90
M15-pCBT-20	-	20	-	80
M15-pCBT-30	-	30	-	70
M25-pCBT-5	-	-	5	95
M25-pCBT-10	-	-	10	90
M25-pCBT-20	-	-	20	80
M25-pCBT-30	-	-	30	70

## Supplementary Materials

The following supporting information can be downloaded at: https://www.mdpi.com/article/10.3390/polym14020323/s1, Figure S1: FE-SEM images of (a) M5, (b) M15, and (c) M25, and atomic force microscopy results of (d) M5, (e) M15, and (f) M25; Figure S2: (a) FT-IR, (b) X-ray photoelectron, and (c) Raman spectra of GNPs; Figure S3: Thermal conductivity of the fabricated composites according to the critical volume fraction based on experimental results; Figure S4: Surface photos of the fabricated composites with M5 (a) 10 wt.% and (b) 30 wt.%; M15 (c) 10 wt.% and (d) 30 wt.%; and M25 (e) 10 wt.% and (f) 30 wt.%; Table S1: In-plane thermal conductivities of composites with GNP fillers; Video S1.
