# Supplementary material for: Thermal Percolation Behavior in Thermal Conductivity of Polymer Nanocomposite with Lateral Size of Graphene Nanoplatelet
Source: Polymers (Basel). 2022 Jan 13;14(2):323. doi: 10.3390/polym14020323 (PMC8780660; doi:10.3390/polym14020323)
Supplement: Supplementary file 1 [file polymers-14-00323-s001.zip › polymers-1543825-supplementary.pdf]

polymers

MDPI

Supplementary Materials

Thermal Percolation Behavior in Thermal Conductivity of Polymer Nanocomposite with Lateral Size of Graphene Nanoplatelet

Ji-un Jang [1,†], Hae Eun Nam [2,†], Soon Oh So [2], Hyeseong Lee [2], Geon Su Kim [2], Seong Yun Kim [2,*] and Seong Hun Kim [1,*]

[1] Department of Organic and Nano Engineering, Hanyang University, 222 Wangsimni-ro, Haengdang-dong, Seongdong-gu, Seoul 04763, Korea; jju204@hanyang.ac.kr (J.-u.J.)

[2] Division of Polymer-Nano & Textile Engineering, Jeonbuk National University, 567 Baekje-daero, Deokjin-gu, Jeonju-si 54896, Jeonbuk, Korea; hae_eun1912@naver.com (H.E.N.); tnsdh0626@naver.com (S.O.S.); dlgptjd6715@naver.com (H.L.); kuean88@naver.com (G.S.K.)

* Correspondence: sykim82@jbnu.ac.kr (S.Y.K.); kimsh@hanyang.ac.kr (S.H.K.); Tel.: +82-63-270-2336 (S.Y.K.); +82-2-2281-0496 (S.H.K.)

† The first two authors contributed equally.

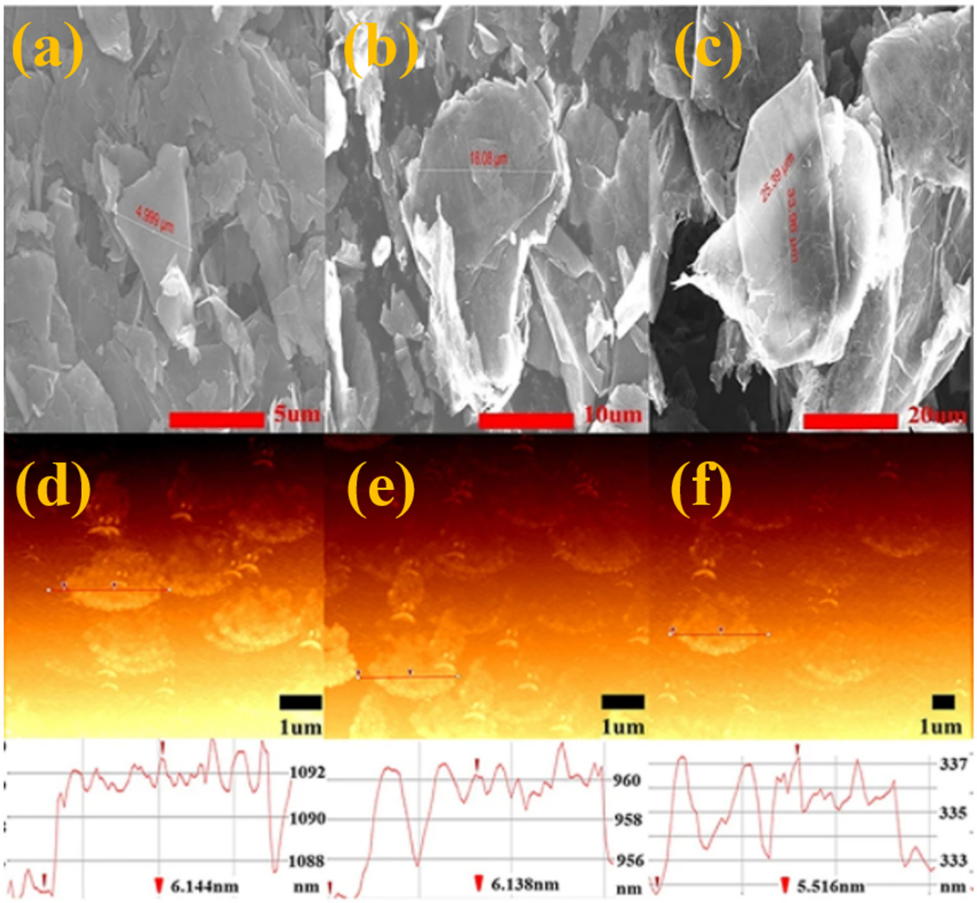

Figure S1. FE-SEM images of (**a**) M5, (**b**) M15 and (**c**) M25, and atomic force microscopy results of (**d**) M5, (**e**) M15 and (**f**) M25 (Reproduced with permission [13]. Copyright 2016, Nature Publishing Group).

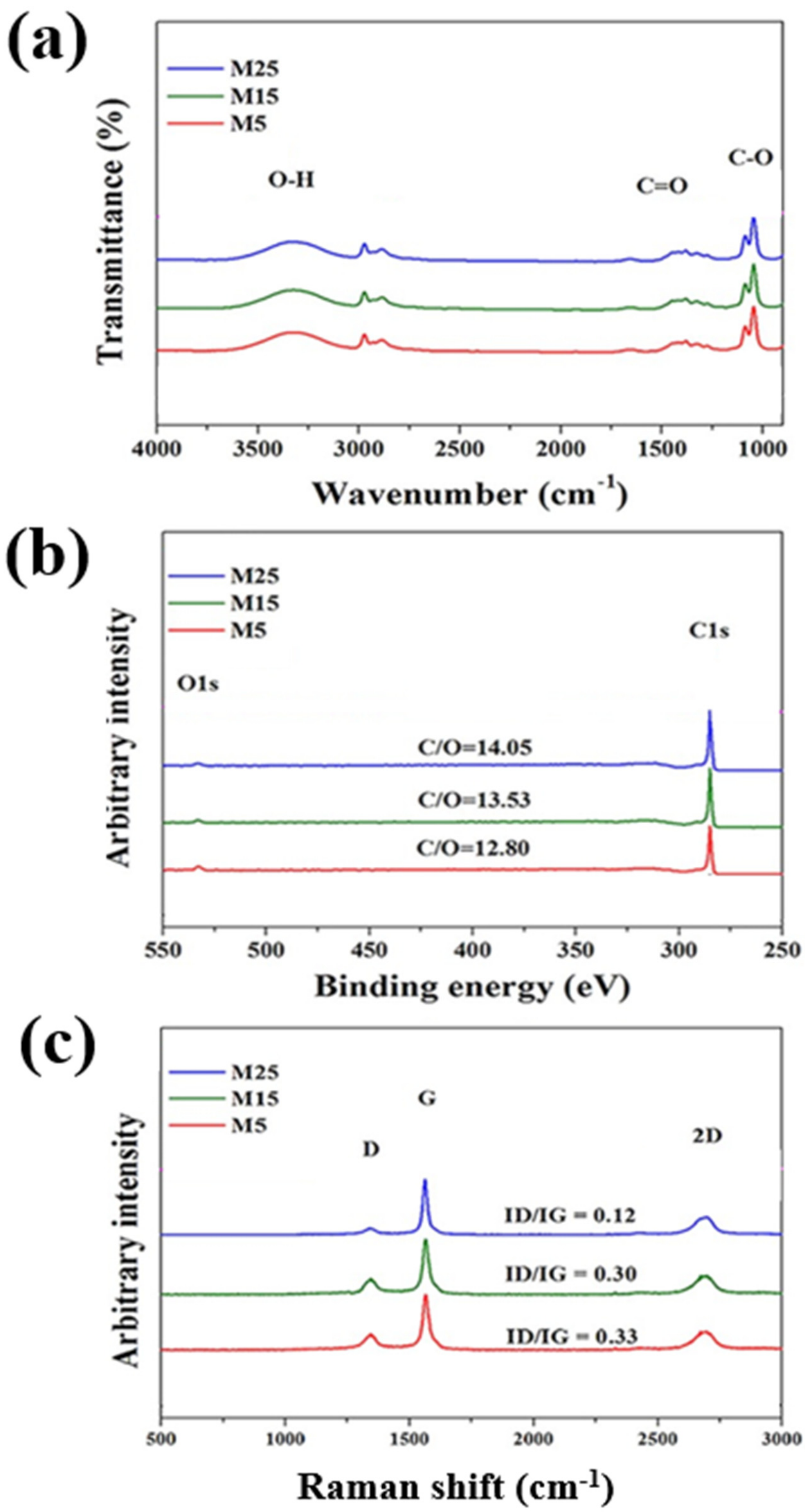

Figure S2. (**a**) FT-IR, (**b**) X-ray photoelectron and (**c**) Raman spectra of GNPs (Reproduced with permission [13]. Copyright 2016, Nature Publishing Group).

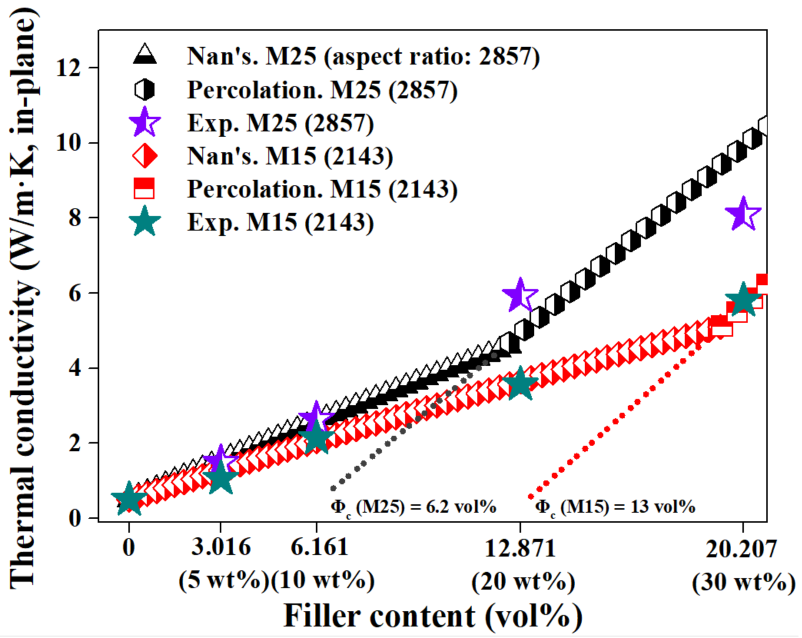

Figure S3. Thermal conductivity of the fabricated composites considering the critical volume fraction based on the experimental results.

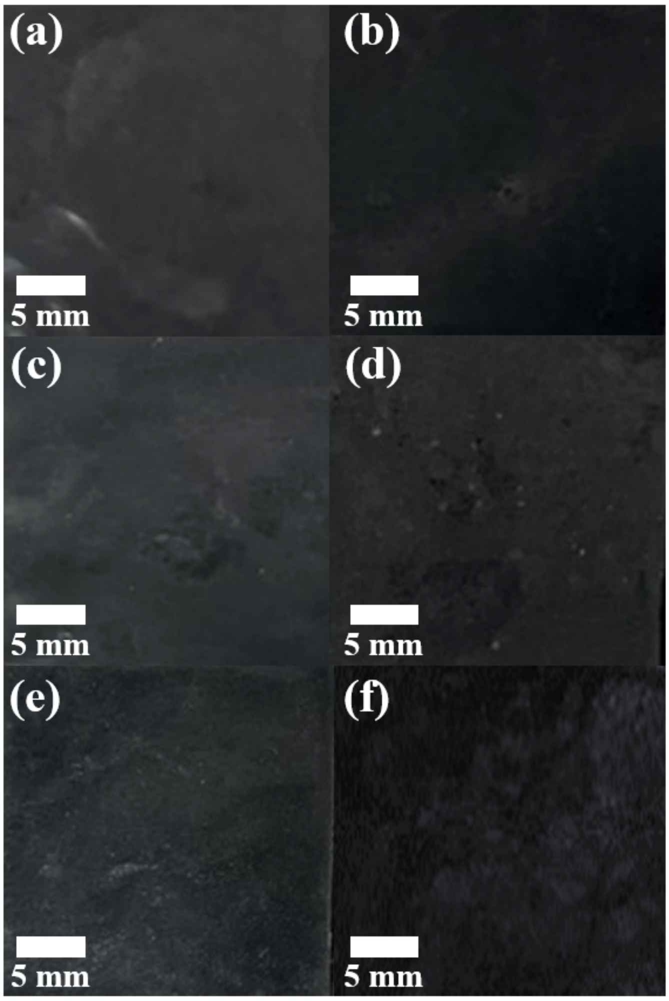

Figure S4. Surface photos of the fabricated composites with M5 (**a**) 10 wt.% and (**b**) 30 wt.%, and M15 (**c**) 10 wt.% and (**d**) 30 wt.%, and M25 (**e**) 10 wt.% and (**f**) 30 wt.%.

Table S1. In-plane thermal conductivities of composites with GNP fillers.

Filler type	In-plane thermal conductivity (W/m·K)	Enhancement (%)	Loading	Matrix	Ref.
GNP (M25)	8.094	1518.8	30 wt.%	pCBT	This work
GNP (M25)	7.10	857	20 wt.%	pCBT	[25]
GNP (M25)	~ 7	~ 3500	50 wt.%	pCBT	[26]
GNP (M25)	~ 6	~ 2500	20 wt.%	pCBT	[13]
GNP (8 μm)	5.82	2430	30 vol.%	PBT/PC	[27]
GNP (M5)	1.98	1320	20 wt.%	pCBT	[22]
GNP (0.5 μm)	1.09	445	20 wt.%	PP	[28]
